# Needle Adjustment Free (NAF) running suture technique (PAN suture) in laparoscopic partial nephrectomy

**DOI:** 10.1186/s12893-021-01112-7

**Published:** 2021-03-06

**Authors:** Jun-wei Pan, Xiang Zhang, Xing-wei Jin, Xiao Liu, Wei-chao Tu, Xian-jin Wang, Bao-xing Huang, Da Xu, Guo-liang Lu, Da-wei Wang, Xiang-hui Wang, Yuan Shao

**Affiliations:** 1 Department of Urology, Ruijin Hospital, Shanghai Jiao Tong University School of Medicine, No. 999, Xi Wang Road, Shanghai, 201801 China; 2 Department of Radiology, Ruijin Hospital, Shanghai Jiao Tong University School of Medicine, Shanghai, China

**Keywords:** Needle Adjustment Free, PAN suture, Needle out‐needle in, Laparoscopic partial nephrectomy, Suture efficiency

## Abstract

**Background:**

It is proposed a new running suture technique called Needle Adjustment Free (NAF) technique, or PAN suture. The efficiency and the safety were evaluated in laparoscopic partial nephrectomy.

**Methods:**

This new running suture technique avoids the Needle Adjustment method used in traditional techniques. The new continuous suture technique (11 patients) was compared with the traditional continuous suture method (33 patients) used in both transperitoneal and retroperitoneal laparoscopic partial nephrectomy (LPN) in terms of suture time (ST), warm ischemia time (WIT), blood loss (BL), open conversion rate and post-op discharge time, post-op bleeding, post-op DVT, ΔGFR (affected side, 3 months post-op). Differences were considered significant when P < 0.05.

**Results:**

ST in the PAN suture group was 30.37 ± 16.39 min, which was significant shorter (P = 0.0011) than in the traditional technique group which was 13.68 ± 3.33 min. WIT in the traditional technique group was 28.73 ± 7.89 min, while in the PAN suture group was 20.64 ± 5.04 min, P = 0.0028. The BL in entirety in the traditional technique group was 141.56 ± 155.23 mL, and in the PAN suture group was 43.18 ± 31.17 mL (P = 0.0017). BL in patients without massive bleeding in the traditional technique group was significantly greater than in the PAN suture group at 101.03 ± 68.73 mL versus 43.18 ± 31.17 mL (P = 0.0008). The open conversion rate was 0 % in both groups. There was no significant difference between the two groups in postoperative discharge time, post-op bleeding, post-op DVT, ΔGFR (affected side, 3 months post-op).

**Conclusions:**

The NAF running suture technique, or PAN suture, leading to less ST, WIT and BL, which was shown to be more effective and safer than the traditional technique used for LPN. A further expanded research with larger sample size is needed.

## Background

Suture efficiency prevents blood loss and aids in recovery of organ function. In laparoscopic surgery, the continuous suture procedure has not proven effective. The reason is that the traditional continuous suture mode, in which Needle In is closely followed by Needle Out, the operator must modulate the needle angle after drawing the thread. Needle Adjustment is time consuming and holds the danger of laparoscope contamination in the process. Thus, to avoid the time consuming aspects of the Needle In-Needle Out technique and to prevent the negative effects of Needle Adjustment, a more efficient procedure is needed.

In this study, we propose a new procedure using a running suture technique called Needle Adjustment Free (NAF). Using this technique, the needle angle does not need to be modulated, thereby yielding greater suture efficiency.

## Methods

### Needle Adjustment Free Technique (PAN suture)

**Fig. 1:**
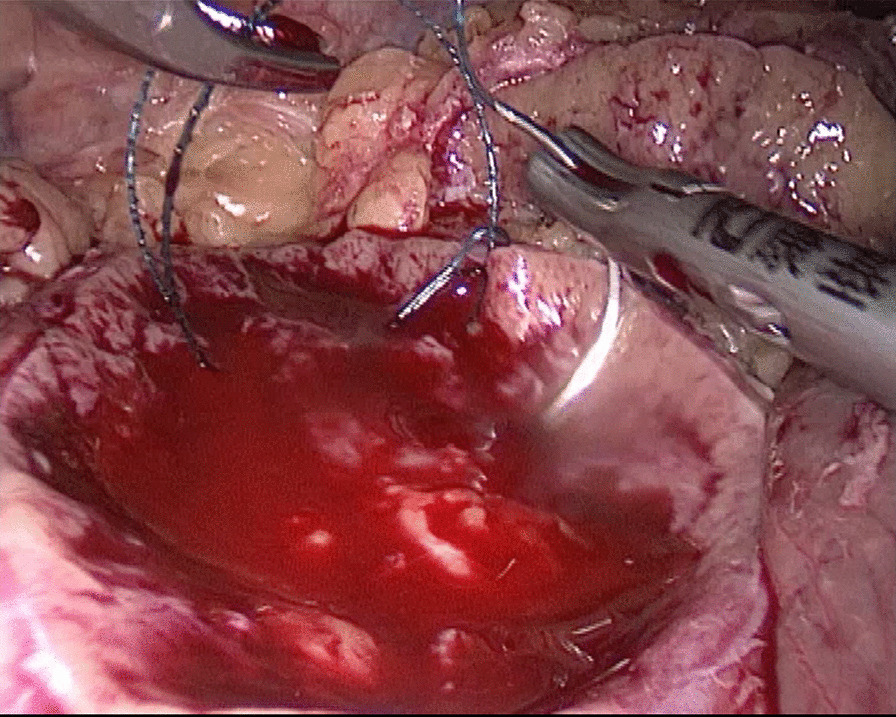
Needle In for the renal wound basement

**Fig. 2:**
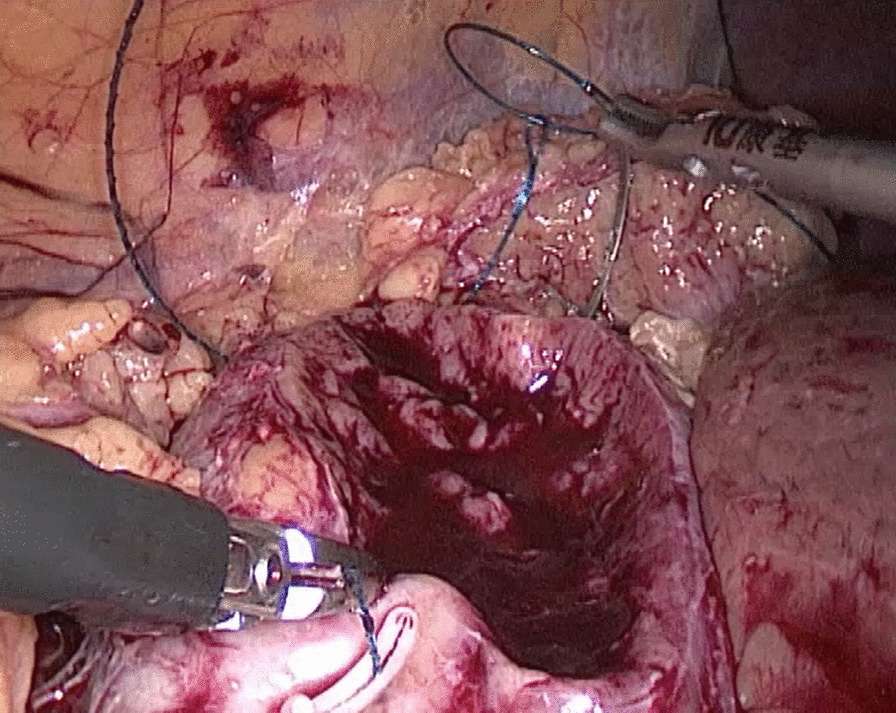
Needle In for the renal outer-layer

**Fig. 3:**
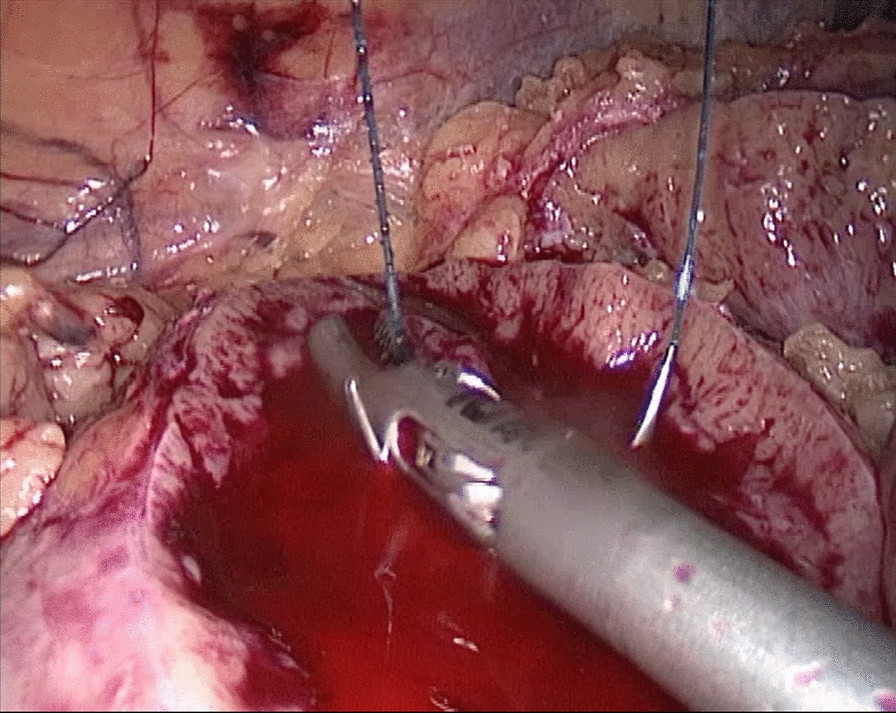
Thread Traction for the renal wound basement

**Fig. 4:**
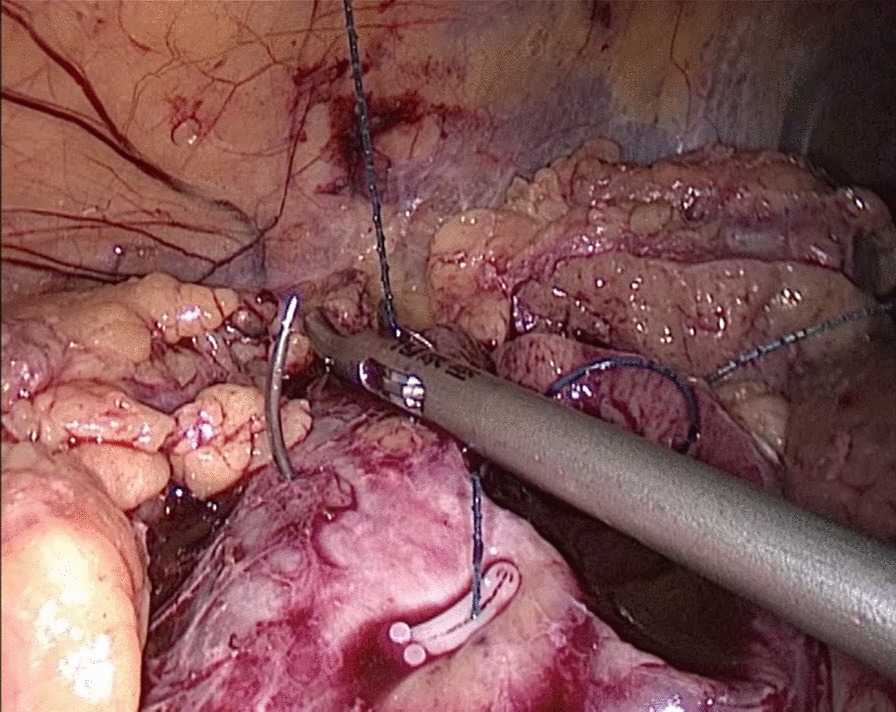
Thread Traction for the renal outer-layer

**Fig. 5:**
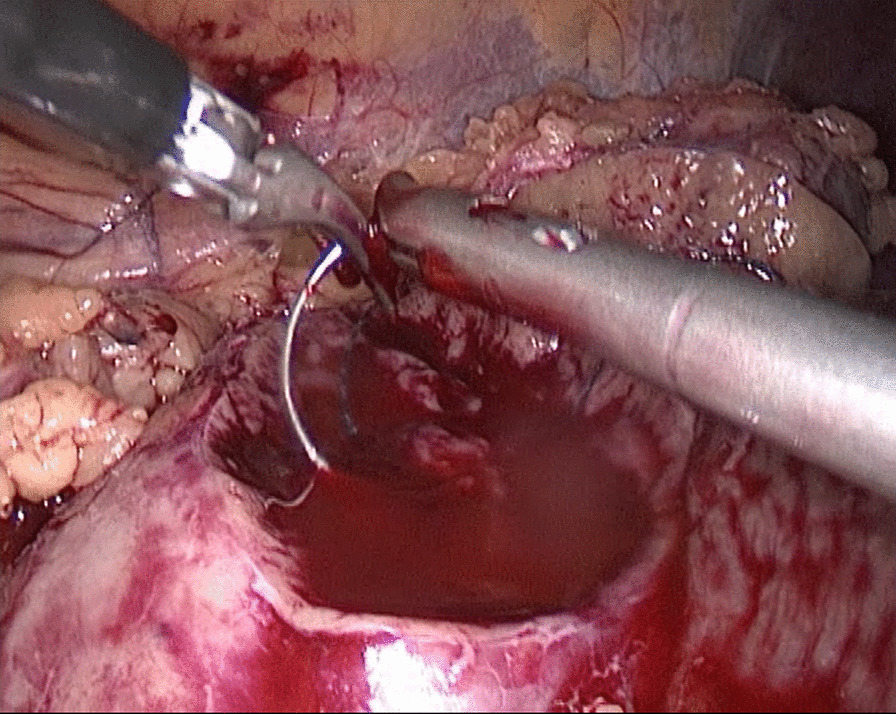
Drawn Out for the renal wound basement

**Fig. 6:**
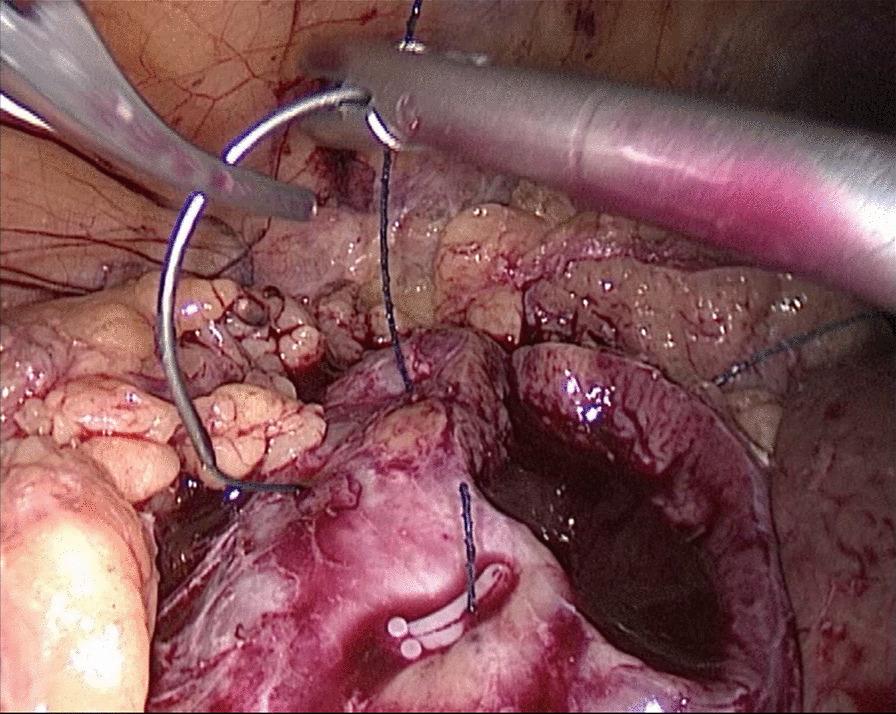
Drawn Out for the renal outer-layer

**Fig. 7:**
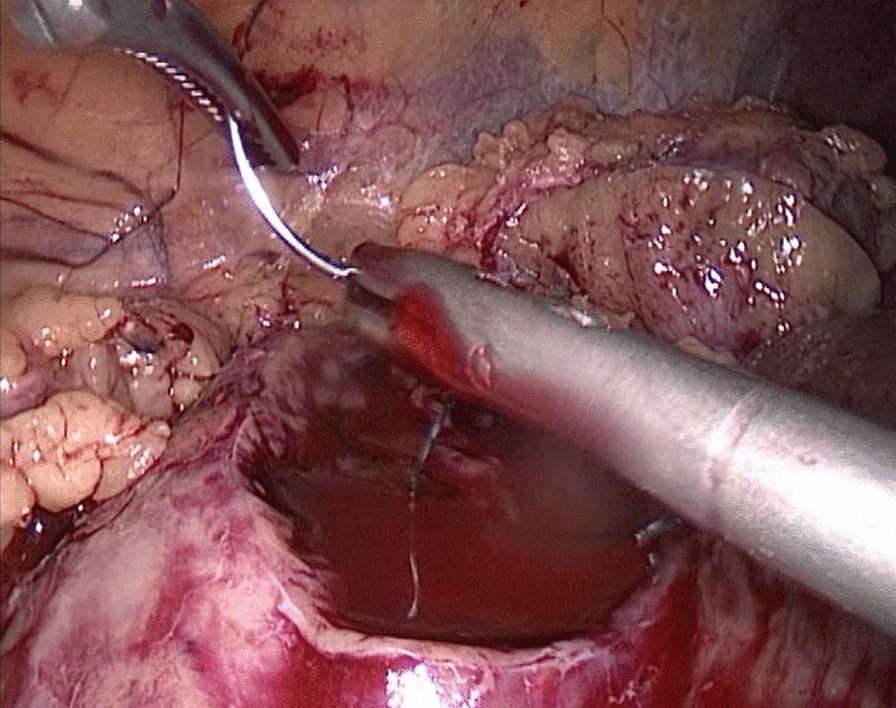
Needle Holding for the renal wound basement

**Fig. 8:**
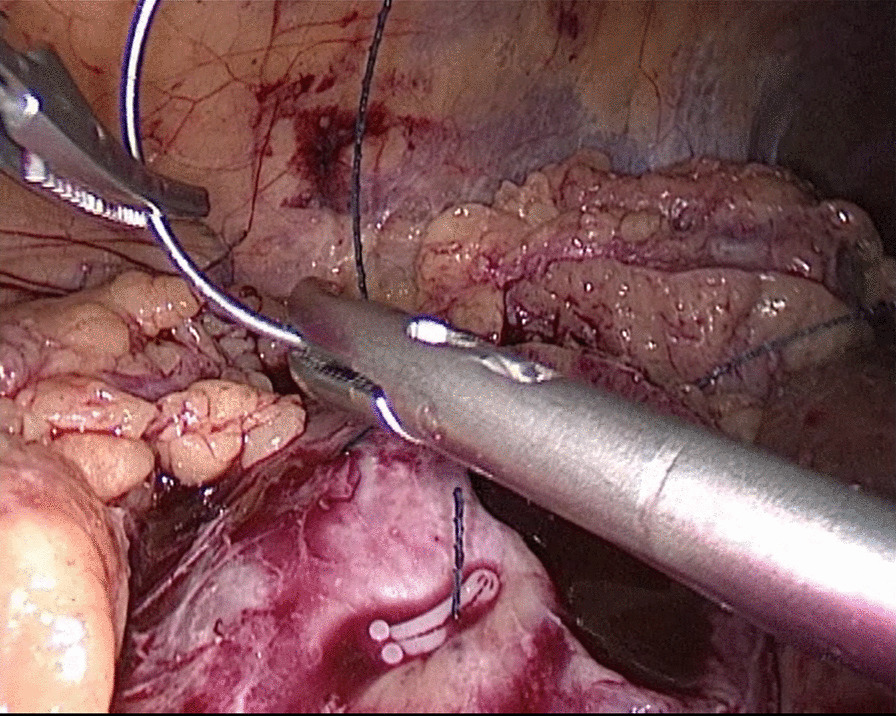
Needle Holding for the renal outer-layer

**Fig. 9:**
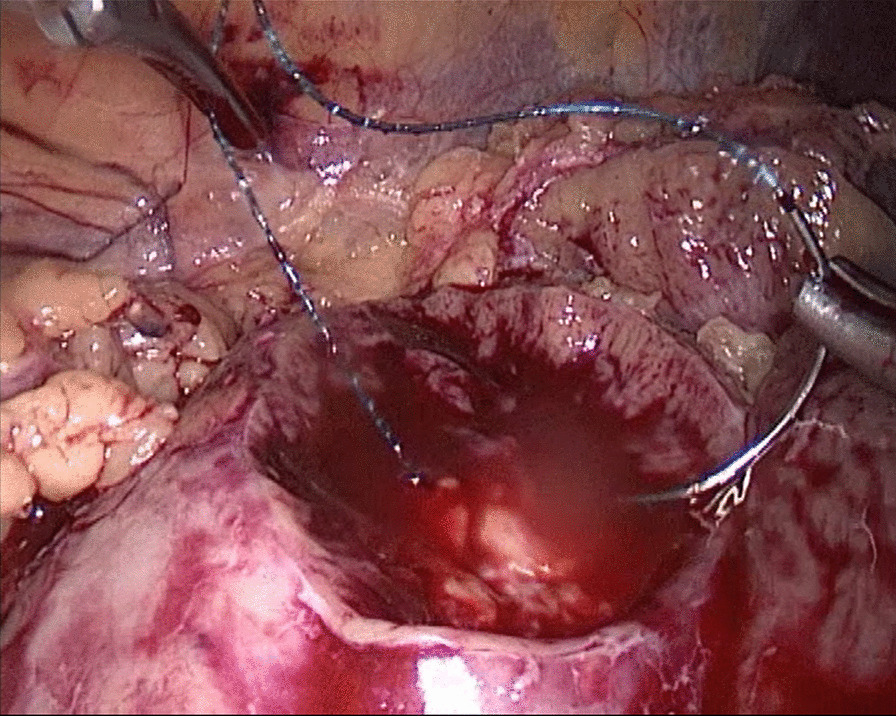
A next Needle In for the renal wound basement

**Fig. 10:**
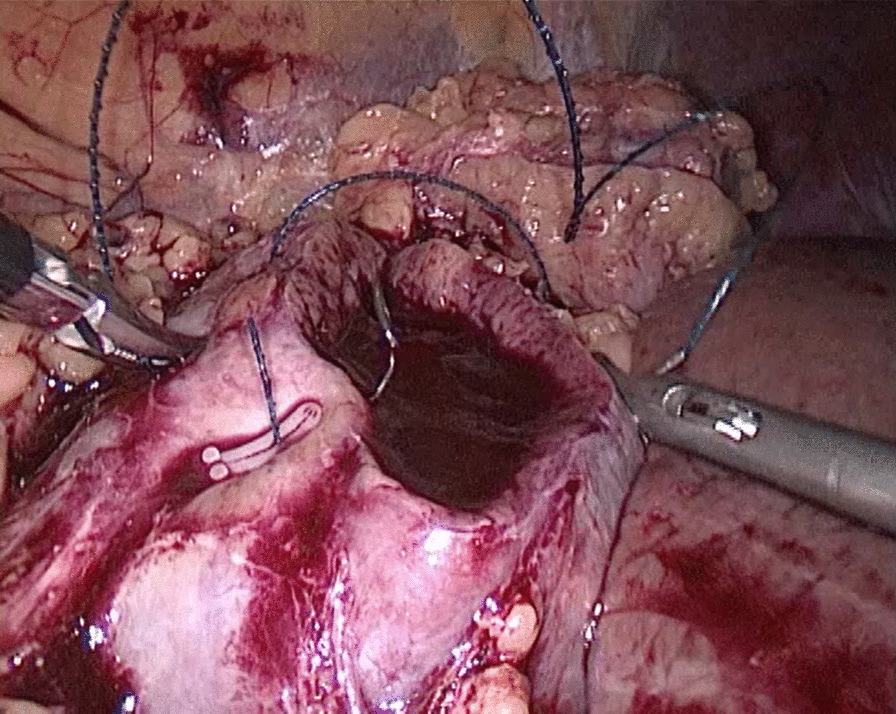
A next Needle In for the renal outer-layer

In the traditional technique, the procedure sequentially follows Needle In, Needle Out, Thread Traction, Needle Adjustment and Needle Holding. However, in the new proposed technique, Needle In is not followed by Needle Out. Conversely, Needle Out is closely followed by Needle In. The process is as follows; in the running suture, after the previous Needle Out, the thread is not pulled tight until another Needle In (Figs. 1, 2). As the needle point breaks through the surface of the tissue, the needle is fixed by the tissue, while the needle angle is not easily changed. Then, a Thread Traction is easily made hand over hand (Figs. 3, 4). Having pulled tight (with or without a Hem-o-Lok™, Ethicon Endo-surgery, Johnson & Johnson, Cincinnati, OH, USA), the suture is then drawn out (Figs. 5, 6) by holding the needle naturally without Needle Adjustment (Figs. 7,  8) to practice a next Needle In (Figs. 9, 10) (Additional file 1: Video S1 NAF in LPN).

For the knot free suture with a ring, such as V-Loc™ (Covidien, Mansfield, MA, USA), the first two stitches can be dealt with by two means: (1) the traditional technique, first Needle In followed by Needle Out, Thread Traction, Needle Adjustment, passing through the ring, Thread re-Traction and Needle re-Adjustment sequentially; (2) PAN suture, first Needle In (until the point breaks through the surface of the tissue) followed by Ring On Point, Needle Out, Needle Holding, another Needle In, Thread Traction, Needle Out, Needle Holding, without any Needle Adjustment. At this stage, it is possible to use the NAF running suture technique, however on occasion the Ring On Point is not possible due to blood accumulation.

### Patient selection

Forty-four patients with kidney neoplasm who were operated on in the manner of LPN (transperitoneal and retroperitoneal) by a single experienced laparoscopic surgeon (JW PAN) in the Department of Urology in Ruijin Hospital from August 2017 to December 2019, were selected in this retrospective study. Thirty-three patients received the traditional technique, while the remaining eleven were treated using the PAN suture.

### Laparoscopic partial nephrectomy

The main renal artery was blocked temporarily by a Bulldog clamp in each operation. The renal wound basement was stitched using a 3 − 0 V-Loc, and the outer-layer was sutured using a 2 − 0 V-Loc. The first two stitches of all sutures were conducted using the traditional technique, careful to consider blood accumulation in the basement. Each suture was consolidated by a Hem-o-Lok™ at the end. The Bulldog clamp was removed once the kidney was reconstructed.

### Parameters

General data including Gender, age, BMI, side, affected side pre-op GFR, approach and R.E.N.A.L. score were gathered in both the traditional technique group and the PAN suture group. These data were subsequently analyzed.

The suture time (ST), warm ischemia time (WIT), blood loss (BL) and post-op discharge time, post-op bleeding, post-op DVT, ΔGFR (3 months post-op) were also recorded and compared between the two groups. BL data were divided into two layers: (1) entire BL, and (2) BL in patients without massive bleeding. This distinction was used since three cases with massive bleeding after using the traditional technique were excluded, taking into consideration the interference of incomplete artery blockage, which could significantly augment the BL. As the bulldog clamp was utilized in all counted cases, the WIT was defined as the time between the placement and the removal of the bulldog.

### Statistical analysis

SAS V8 was used to analyze the data. The data were presented as mean ± standard error of the mean (SEM). Homogeneity test of variance, group *t*-test, Chi-square test of a fourfold table and Fischer exact test were used to analyze the differences between the two groups. Differences were considered significant when P < 0.05.

### Statement of IRB approval

This study was approved by Ruijin Hospital Ethics Committee and the informed consent was allowed to be exempted.

## Results

### General data statistics

General data showed no significant difference between the traditional technique group (n = 33) and the PAN suture group (n = 11) (Table 1).

**Table 1:** General data statistics in the traditional technique group and the PAN suture group

Parameters		Traditional technique group (n = 33)	PAN suture group (n = 11)	P value
Gender	Male (n, %)	14 (42.42)	8 (72.73)	0.1623
	Female (n, %)	19 (57.58)	3 (27.27)	
Age (years)		55.03 ± 13.83	49.21 ± 16.94	0.2242
BMI (kg/m^2^)		23.73 ± 3.19	24.02 ± 3.05	0.7979
Side	Left (n, %)	17 (51.52)	4 (36.36)	0.4941
	Right (n, %)	16 (48.48)	7 (63.64)	
Affected side pre-op GFR		42.774 ± 12.307	43.277 ± 12.422	0.9267
Approach	Transperitoneal (n,%)	6 (18.18)	1 (9.09)	0.6594
	Retroperitoneal (n,%)	27 (81.82)	10 (90.91)	
R.E.N.A.L. score (points)		6.24 ± 1.58	5.82 ± 1.17	0.4192

*BMI* body mass index, *GFR *glomerular filtration rate

### ST, WIT, BL and post‐op discharge time

ST in the PAN suture group was 30.37 ± 16.39 min, which was significantly shorter (P = 0.0011) than in the traditional technique group at 13.68 ± 3.33 min. WIT in the traditional technique group was 28.73 ± 7.89 min, while in the PAN suture group was 20.64 ± 5.04 min (P = 0.0028). For BL there were two comparisons: entire BL and BL in patients without massive bleeding. This is due to three excessive bleeding patients that were excluded from the traditional technique group, taking consideration of incomplete blockage. The entire BL in the traditional technique group was 141.56 ± 155.23 mL, and in the PAN suture group was 43.18 ± 31.17 mL (P = 0.0017). The BL in patients with no massive bleeding in the traditional technique group was significantly greater than in the PAN suture group at 101.03 ± 68.73 mL versus 43.18 ± 31.17 mL (P = 0.0008). The open conversion rate was 0 % in both groups. There were no significant differences between the two groups in post-operative discharge time (6.69 ± 1.83 days and 6.27 ± 1.13 days, respectively, P = 0.2645), post-op bleeding (0.00 % and 0.00 %), post-op DVT (0.00 % and 0.00 %), ΔGFR (affected side, 3 months post-op) (12.18 ± 5.25 and 10.58 ± 6.00, P = 0.7374). (Table 2)

**Table 2:** Comparison between the traditional technique group and the PAN suture group

Parameters	Traditional technique group (n = 33)	PAN suture group (n = 11)	P value
ST (min)	30.37 ± 16.39	13.68 ± 3.33	0.0011
WIT (min)	28.73 ± 7.89	20.64 ± 5.05	0.0028
BL entirely (mL)	141.56 ± 155.23	43.18 ± 31.17	0.0017
BL in no massive bleeding patients (3 excluded from traditional technique group)	101.03 ± 68.73	43.18 ± 31.17	0.0008
Open conversion (n, %)	0 (0.00)	0 (0.00)	–
Post-op discharge time (d)	6.69 ± 1.83	6.27 ± 1.13	0.2645
Post-op bleeding (n, %)	0 (0.00)	0 (0.00)	–
Post-op DVT (n, %)	0 (0.00)	0 (0.00)	–
ΔGFR (affected side, 3 months post-op) (mL/min)	12.18 ± 5.25	10.58 ± 6.00	0.7374

*ST *suture time, *WIT *warm ischemia time, *BL *blood loss, *DVT *deep venous thrombosis, ΔGFR = pre-op GFR – post-op GFR

## Discussion

Suture efficiency is a crucial step in preservation of organ function, blood loss, and even the patient’s life. For example, in partial nephrectomy, when the kidney is in hot ischemia after temporary occlusion of the main renal artery, the suture time (ST) of the wound due to the tumorectomy is closely related to the preservation of renal function on the affected side [1]. Similarly, in upper abdominal surgery, inferior vena cava damage will lead to bleeding, and the suture time of repairing the venous rupture will be directly or indirectly related to the blood loss.

Therefore, various kinds of suture methods, materials, and instruments have emerged, such as the continuous interlocking technique, Lembert technique, Connell technique, Gambia technique and Zipper technique for running suture of the cavity organ wall [2]. Likewise, the single needle continuous suture method has been used in urethro-bladder neck anastomosis in radical prostatectomy [3] and the running suture technique in laparoscopic nephron sparing nephrectomy [1, 4]. Several studies have also reported barbed sutures such as V-Loc™ and simplified sutures methods which benefited the surgery [5–8]. Finally, the automatic stapler (ex. Ethicon^®^ circular stapler) and auto-suture (ex. Autosuture™, Endo Stitch™, SILS Stitch™) have also been reported [9–11]. All of the above techniques significantly improved suture efficiency, but each has their own limitations. In surgery, especially in laparoscopic surgery, where suture activities cannot be replaced by automatic instruments, suture efficiency is of great importance.

Rather than wait for future automatic instruments, we re-evaluated the classic continuous suture technique. This traditional method was deconstructed into a circulation sequence of Needle In, Needle Out, Thread Traction, Needle Adjustment and Needle Holding, in which Needle In-Needle Out is a fixed process. Although this is the accepted procedure, it inevitably leads to repeated Thread Traction and harmful Needle Adjustments. In open surgery, such a traditional method does not cause much time loss, especially when there is an assistant to help with Thread Traction. However, in laparoscopic surgery, repeated Thread Traction and Needle Adjustment significantly affects suture efficiency. This is due in part to the lack of an assistant, and the more important harmful effects of the Needle Adjustment. Also, under the laparoscope, the space the needle is placed in after Thread Traction is not where the stitched tissue is located. This fixed process is inevitably accompanied by the movement of laparoscope, and includes further contamination of the camera lens. The higher the frequency of laparoscope movement, the higher the probability of lens pollution. During surgery, when there is a sudden serious active bleeding, the contamination caused by the camera lens could be fatal. In addition, in the retroperitoneal adrenal area, the pelvic floor or other limited spaces, every time the lens of the laparoscope is stained, the emotion of the operator may also be disturbed.

For those trained in laparoscopic surgery, it is difficult to distinguish distance and adjusting the needle, especially during needle adjustment. Therefore, reducing the need of Needle Adjustment can significantly improve the fluency of the operation and self-confidence of beginners in the technique, while also conserving physical strength. In order to avoid needle displacement and Needle Adjustment following Thread Traction in the traditional mode, a new technique of Needle Adjustment Free (NAF) is proposed. This technique modulates the order of Needle In, Needle Out and Thread Traction, and makes the needle fixed by the stitched tissue. Using this technique, Needle Adjustment and the laparoscope movement are avoided thereby increasing suture efficiency. In the present study, the ST and WIT in the PAN suture group were significant shorter than in the traditional technique group, (P = 0.0011 and 0.0028, respectively), revealing the higher suture efficiency of the NAF running suture technique. The entire BL and the BL in patients without massive bleeding in the traditional technique group was significantly greater than in the PAN suture group, (P = 0.0017 and 0.0008, respectively). This indicated both a higher suture efficiency and a higher safety in the NAF running suture technique. There was not statistical significance between the two groups in comorbidities. These results implied that the novel running suture technique has a greater advantage in LPN. This technique has already been successfully used in laparoscopic diverticulectomy of the bladder, bladder neck plasty and inferior vena cava repair, by the same operator (JW PAN). With additional further research, the PAN suture may have a more widespread clinical application and may replace traditional techniques in laparoscopic surgery.

## Supplementary Information

**Additional file 1**.  NAF in LPN.

## Data Availability

The datasets used and/or analysed during the current study are available from the corresponding author on reasonable request.

## Abbreviations

NAF: Needle Adjustment Free
LPN: Laparoscopic partial nephrectomy
ST: Suture time
WIT: Warm ischemia time
BL: Blood loss
DVT: Deep venous thrombosis
GFR: Glomerular filtration rate
BMI: Body mass index

## References

[1] Klatte T, Ficarra V, Gratzke C, Kaouk J, Kutikov A, Macchi V, Mottrie A, Porpiglia F, Porter J, Rogers CG, Russo P, Thompson RH, Uzzo RG, Wood CG, Gill IS (2015). A literature review of renal surgical anatomy and surgical strategies for partial nephrectomy. Eur Urol.

[2] Haksever M, Akduman D, Aslan S, Solmaz F, Ozmen S (2015). Modified continuous mucosal connell suture for the pharyngeal closure after total laryngectomy: zipper suture. Clin Exp Otorhinolaryngol.

[3] Yang J, Shao PF, Lv Q, Song NH, Li J, Zhang W, Li P, Hua LX, Yin CJ (2014). Continuous suture of a single absorbable suture: a new simplified vesicourethral anastomosis technique in laparoscopic radical prostatectomy. Int Surg.

[4] Kaygisiz O, Çelen S, Vuruşkan BA, Vuruşkan H (2017). Comparison of two different suture techniques in laparoscopic partial nephrectomy. Int Braz J Urol.

[5] Canales BK, Lynch AC, Fernandes E, Anderson JK, Ramani AP (2007). Novel technique of knotless hemostatic renal parenchymal suture repair during laparoscopic partial nephrectomy. Urology.

[6] Ye J, Zhang S, Tian X, Wang G, Zhao L, Ma L (2018). Knotless retroperitoneoscopic nephron-sparing surgery for small renal masses: comparison of bipolar sutureless technique and barbed suture technique. J Int Med Res.

[7] Lin Y, Liao B, Lai S, Huang J, Du L, Wang K, Li H (2019). The application of barbed suture during the partial nephrectomy may modify perioperative results: a systematic review and meta-analysis. BMC Urol.

[8] Rong S, Lewis AG, Kunter U, Haller H, Gueler F. A knotless technique for kidney transplantation in the mouse. J Transplant. 2012;2012:127215. 10.1155/2012/127215 PMC3407654 22852070

[9] Adams JB, Schulam PG, Moore RG, Partin AW, Kavoussi LR (1995). New laparoscopic suturing device: Initial clinical experience. Urology.

[10] Göpel T, Härtl F, Schneider A, Buss M, Feussner H (2011). Automation of a suturing device for minimally invasive surgery. Surg Endosc.

[11] Kurenov SN, Punak S, Kim M, Peters J, Cendan JC (2006). Simulation for training With the Autosuture™ Endo Stitch™ Device. Surg Innov.

